# Radiotherapy in Li-Fraumeni Syndrome: From Biological Concern to Personalized Clinical Decision-Making

**DOI:** 10.7759/cureus.100978

**Published:** 2026-01-07

**Authors:** Reyzane El Mjabber, Rim Alami, Nada Filali, Abderrahim Bourial, Zineb Dahbi, Fadila Kouhen, Nabil Ismaili, Sanaa El Majjaoui, Asmaa Naim

**Affiliations:** 1 Radiation Oncology, Mohammed VI University of Health Sciences, Casablanca, MAR; 2 Radiation Oncology, Cheikh Khalifa International University Hospital, Casablanca, MAR; 3 Medical Oncology, Mohammed VI University of Health Sciences, Casablanca, MAR; 4 Medical Oncology, Cheikh Khalifa International University Hospital, Casablanca, MAR; 5 Otolaryngology, Mohammed VI University of Health Sciences, Casablanca, MAR; 6 Otolaryngology, Cheikh Khalifa International University Hospital, Casablanca, MAR; 7 Radiation Oncology, Mohammed VI International University Hospital, Rabat, MAR

**Keywords:** li-fraumeni syndrome, radiation-induced malignancies, radiotherapy, secondary malignancies, tp53 germline mutation

## Abstract

Li-Fraumeni syndrome (LFS) is an inherited cancer predisposition syndrome caused by germline pathogenic variants in *TP53*. It is characterized by a high risk of developing malignancies throughout life, often at an early age. Because p53 is involved in cellular responses to DNA damage, the use of radiotherapy in this population has raised long-standing clinical concerns regarding potential late effects. This narrative review outlines key biological considerations related to radiation exposure in *TP53* mutation carriers and summarizes the clinical experience reported with radiotherapy in patients with LFS. From a biological perspective, altered p53 function may influence how cells respond to ionizing radiation and how damaged cells are managed. These considerations have led to caution when considering radiotherapy in this setting. From a clinical perspective, the available literature remains limited and largely retrospective. Published reports describe variable outcomes after radiotherapy, including cases of secondary malignancies developing within previously irradiated regions, as well as cases without apparent long-term complications. Overall, clinical responses and long-term outcomes appear heterogeneous. In the absence of prospective data, radiotherapy in LFS should not be viewed as an absolute contraindication. Its use may be considered in selected clinical situations after careful multidisciplinary discussion. Decisions should balance the expected oncologic benefit with potential long-term risks and available alternatives. When radiotherapy is used, careful treatment planning to limit normal tissue exposure and long-term follow-up are essential components of patient care.

## Introduction and background

Li-Fraumeni syndrome (LFS) is a hereditary cancer predisposition disorder associated with a very high lifetime risk of malignancy [1]. Clinically, LFS is notable for its broad tumor spectrum and early age of onset. It is mainly caused by germline pathogenic variants in the TP53 gene, which encodes the tumor suppressor protein p53. Molecular analyses have identified many distinct pathogenic TP53 variants in patients with LFS [2]. Most inherited mutations affect the DNA-binding domain of p53 and impair its transcriptional activity. In some cases, these variants not only result in loss of function but also interfere with the remaining wild-type protein or acquire oncogenic gain-of-function properties, further increasing cancer risk [3].

Under normal conditions, p53 protects cells from genomic damage by inducing cell-cycle arrest, activating DNA repair, or triggering programmed cell death. When this pathway is disrupted, cells can continue to divide despite accumulating genetic alterations, which facilitates malignant transformation [3].

In fact, malignancies of the breast, central nervous system, adrenal cortex, bone, soft tissue, and hematopoietic system are frequently encountered, and affected individuals often develop more than one primary cancer during their lifetime. The cumulative probability of developing cancer increases rapidly with age, particularly in women, and is further compounded by exposure to therapeutic interventions that induce DNA damage [4].

Radiotherapy remains a cornerstone of cancer management, with a substantial proportion of adult patients receiving radiation as part of curative or palliative treatment strategies. In contrast, its application in patients with LFS has historically been restrained due to concerns about long-term safety and the absence of robust prospective data. The rarity of the syndrome and the tendency to avoid radiation exposure have limited the generation of high-quality evidence, leaving clinicians without clear, evidence-based guidance.

In the general oncology population, the development of radiation-associated secondary malignancies is relatively uncommon. However, in patients with hereditary cancer predisposition syndromes, including LFS, this risk appears disproportionately elevated. This observation has had a significant influence on treatment decision-making, particularly with regard to the use of radiotherapy [5].

In this context, the present review aims to clarify the biological rationale underlying concerns about radiotherapy in LFS, to summarize the available clinical evidence, and to propose a pragmatic framework to support individualized clinical decision-making, with particular attention to subsequent malignancy risk and survival outcomes.

## Review

Why radiotherapy raises specific concerns in LFS

Radiotherapy exerts its antitumor effect primarily by inducing DNA damage, including single- and double-strand breaks, which are usually sensed and repaired through p53-dependent cellular pathways. In individuals with LFS, germline TP53 mutations disrupt these protective mechanisms, potentially altering normal tissue responses to ionizing radiation. Impaired cell-cycle checkpoint control, defective DNA repair signaling, and reduced elimination of damaged cells may allow radiation-exposed cells to survive despite persistent genomic instability. This altered response creates a biological context in which radiation-induced mutations may be more readily fixed and propagated, thereby increasing the theoretical risk of malignant transformation in normal tissues [6].

In addition to compromised apoptotic and senescence pathways, TP53 dysfunction may influence the balance between error-free and error-prone DNA repair processes following radiation exposure. In the absence of effective p53-mediated surveillance, cells with misrepaired DNA lesions may escape elimination and continue to proliferate. Over time, the accumulation of such lesions can contribute to chromosomal instability and oncogenic evolution. These mechanisms provide a biologically plausible explanation for the heightened concern regarding radiation-associated secondary malignancies in patients with LFS, particularly in long-term survivors treated at a young age [7].

Preclinical investigations have consistently shown that loss of functional p53 markedly modifies cellular responses to ionizing radiation. Studies employing TP53-deficient cell lines and genetically engineered animal models demonstrate impaired activation of radiation-induced apoptosis, defective enforcement of cell-cycle checkpoints, and inappropriate survival of cells harboring radiation-induced genomic lesions [8]. Importantly, this phenotype should not be interpreted as therapeutic radioresistance; rather, it reflects a failure of damage surveillance mechanisms, resulting in persistence of genetically unstable cells and an increased mutational burden within irradiated tissues [9].

Translational observations further corroborate these experimental findings. Molecular analyses of radiation-associated malignancies have identified genomic signatures indicative of defective DNA damage response pathways, including chromosomal instability and complex structural rearrangements [10]. In the setting of germline TP53 mutations, such alterations suggest that ionizing radiation may function as a facilitating factor in tumor initiation by permitting the survival and expansion of damaged cells, rather than acting as a direct and isolated carcinogenic event [11]. Notably, current evidence does not support a uniform or deterministic risk associated with radiotherapy in all TP53 mutation carriers. Instead, it highlights a context-dependent biological response to radiation exposure, underscoring the need for individualized risk-benefit assessment when radiotherapy represents a potential component of treatment, particularly in scenarios where alternative local modalities are limited or suboptimal.

In summary, TP53 is a key regulator of DNA damage responses, including repair, apoptosis, and senescence. Germline TP53 dysfunction in LFS alters cellular responses to ionizing radiation, underscoring the need for a cautious and individualized approach to radiotherapy.

Biological rationale

TP53 in DNA Damage Response

Upon the detection of DNA damage, p53 is stabilized and activated through post-translational modifications mediated by upstream signaling pathways, notably the ATM and ATR kinases [12]. Activated p53 subsequently induces transcription of cell-cycle regulatory genes, most prominently CDKN1A (p21), resulting in cell-cycle arrest and the establishment of a temporal window that allows DNA repair mechanisms to operate. Through this checkpoint control, p53 restricts the propagation of cells harboring unrepaired or misrepaired genomic lesions [13].

In parallel, p53 directly influences multiple DNA repair pathways. It modulates nucleotide excision repair, base excision repair, and homologous recombination by regulating the expression and activity of repair-associated proteins. This regulatory role extends beyond transcriptional control, as p53 also interacts directly with components of the DNA repair machinery, thereby contributing to the maintenance of genomic fidelity. When repair is successful, normal cell-cycle progression may resume; however, persistent or excessive DNA damage shifts p53 signaling toward irreversible cellular outcomes.

Apoptosis represents one such outcome and constitutes a critical tumor-suppressive mechanism mediated by p53. In response to severe genomic insult, p53 promotes programmed cell death by upregulating pro-apoptotic genes such as BAX, PUMA, and NOXA, while simultaneously repressing survival pathways. This process ensures the elimination of cells with high oncogenic potential, thereby preventing clonal expansion of genetically unstable cells. The balance between DNA repair and apoptosis is tightly regulated and depends on both the extent of damage and the cellular context [14].

Cellular senescence provides an additional protective mechanism governed by p53 activity. Senescence is characterized by permanent cell-cycle arrest accompanied by metabolic and transcriptional reprogramming. By inducing sustained expression of cell-cycle inhibitors and altering chromatin organization, p53-driven senescence acts as a barrier to malignant transformation in cells that have experienced oncogenic stress or DNA damage. Although senescent cells remain viable, their inability to proliferate contributes to long-term tumor suppression [15].

Consequences of Germline TP53 Pathogenic Variants

Collectively, these interconnected functions position TP53 as a key determinant of cellular fate following genomic injury. Disruption of p53 signaling compromises DNA repair capacity, impairs apoptotic clearance, and weakens senescence induction, thereby facilitating tumor initiation and progression-an effect that is particularly pronounced in hereditary cancer predisposition syndromes such as LFS [7].

In addition to germline TP53 pathogenic variants, interindividual differences in cancer risk among patients with LFS are further shaped by genetic and epigenetic modifiers. These include functional polymorphisms within TP53 and its regulatory network, such as TP53 p.Arg72Pro (rs1042522), MDM2 SNP309 (c.14+309T>G; rs2279744), and MIR605 n.74T>C (rs2043556). Additional modifiers include the TP53 intron 3 duplication (PIN3; rs17878362), truncating variants in XAF1 (p.Glu134Ter), telomere shortening across generations, and emerging epigenetic modifiers involving WNT signaling and inherited epimutations in ASXL1, ETV6, and LEF1 [2].

Preclinical and Translational Evidence

Radiotherapy exerts its antitumor effect primarily through the induction of DNA damage, including single- and double-strand breaks, which are normally sensed and resolved through p53-dependent cellular pathways. In individuals with LFS, germline alterations in TP53 disrupt these protective mechanisms, potentially altering normal tissue responses to ionizing radiation. Impaired cell-cycle checkpoint control, defective DNA repair signaling, and reduced elimination of damaged cells may allow radiation-exposed cells to survive despite persistent genomic instability. This altered response creates a biological context in which radiation-induced mutations may be more readily fixed and propagated, thereby increasing the theoretical risk of malignant transformation in normal tissues [6].

In addition to compromised apoptotic and senescence pathways, TP53 dysfunction may influence the balance between error-free and error-prone DNA repair processes following radiation exposure. In the absence of effective p53-mediated surveillance, cells with misrepaired DNA lesions may escape elimination and continue to proliferate. Over time, the accumulation of such lesions can contribute to chromosomal instability and oncogenic evolution. These mechanisms provide a biologically plausible explanation for the heightened concern regarding radiation-associated secondary malignancies in patients with LFS, particularly in long-term survivors treated at a young age [7].

Preclinical investigations have consistently shown that loss of functional p53 markedly modifies cellular responses to ionizing radiation. Studies employing TP53-deficient cell lines and genetically engineered animal models demonstrate impaired activation of radiation-induced apoptosis, defective enforcement of cell-cycle checkpoints, and inappropriate survival of cells harboring radiation-induced genomic lesions [8]. Importantly, this phenotype should not be interpreted as therapeutic radioresistance; rather, it reflects a failure of damage surveillance mechanisms, resulting in persistence of genetically unstable cells and an increased mutational burden within irradiated tissues [9].

Translational observations further corroborate these experimental findings. Molecular analyses of radiation-associated malignancies have identified genomic signatures indicative of defective DNA damage response pathways, including chromosomal instability and complex structural rearrangements [10]. In the setting of germline TP53 mutations, such alterations suggest that ionizing radiation may function as a facilitating factor in tumor initiation by permitting the survival and expansion of damaged cells, rather than acting as a direct and isolated carcinogenic event [11]. Notably, current evidence does not support a uniform or deterministic risk associated with radiotherapy in all TP53 mutation carriers. Instead, it highlights a context-dependent biological response to radiation exposure, underscoring the need for individualized risk-benefit assessment when radiotherapy represents a potential component of treatment, particularly in scenarios where alternative local modalities are limited or suboptimal.

**Figure 1:**
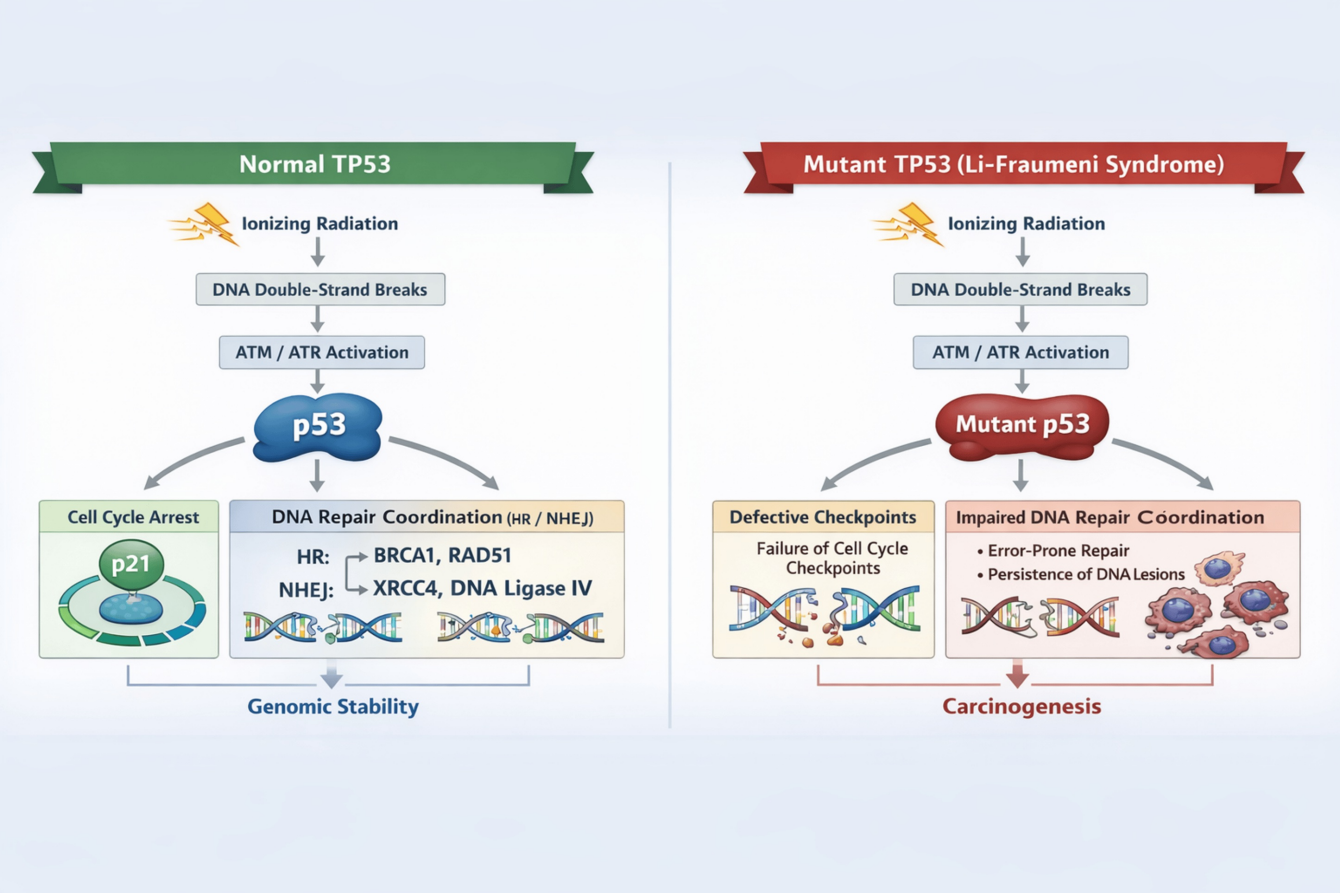
TP53-mediated cellular response to radiation-induced DNA damage in normal cells and Li-Fraumeni syndrome. HR: homologous recombination, ATM: ataxia-telangiectasia mutated, ATR: ataxia telangiectasia and Rad3-related, BRCA1: breast cancer 1, RAD51: RAD51 recombinase, XRCC4: X-ray repair cross-complementing protein 4, NHEJ: non-homologous end joining This figure was created using ChatGPT (OpenAI, San Francisco, California).

In summary, TP53 is a key regulator of DNA damage responses, including repair, apoptosis, and senescence. Germline TP53 dysfunction in LFS alters cellular responses to ionizing radiation, supporting a cautious and individualized approach to radiotherapy (Figure 1).

What does clinical evidence actually show?

Case Reports

Clinical evidence on the use of radiotherapy in patients with LFS is predominantly derived from individual case reports, which provide detailed but heterogeneous descriptions of treatment exposure and subsequent outcomes. Several reports describe the occurrence of secondary malignancies arising within previously irradiated fields, often after relatively short latency periods. These cases frequently involve high-grade sarcomas, including angiosarcoma and osteosarcoma, and typically occur in young patients with confirmed germline TP53 mutations.

Kondapavulur et al. [16] described two patients with genetically confirmed LFS who developed secondary cranial sarcomas following radiotherapy for primary brain tumors. One patient developed a pleomorphic sarcoma of the skull four years after chemoradiotherapy for a low-grade astrocytoma, while the second developed an osteosarcoma five years after proton beam therapy for a high-grade glioma. These cases illustrate the development of secondary sarcomas within irradiated fields after relatively short latency periods and suggest that even highly conformal radiation techniques do not fully eliminate secondary cancer risk in TP53 mutation carriers. However, the descriptive nature of case reports precludes definitive causal inference.

A representative example is provided by Barbosa et al. [17], who reported a young woman with LFS treated with breast-conserving surgery followed by adjuvant chemotherapy and radiotherapy for invasive ductal carcinoma of the breast. Several years after irradiation, the patient developed an epithelioid angiosarcoma arising in the previously irradiated breast. This case illustrates a classical presentation of radiation-associated sarcoma in a genetically predisposed host and highlights the clinical dilemma posed by standard radiotherapy indications in patients with hereditary cancer susceptibility syndromes. While such reports support biological plausibility for increased radiation-associated risk in LFS, their descriptive nature and the absence of comparator groups preclude definitive causal inference.

García Novoa et al. [18] reported a 37-year-old woman with a strong familial history of early-onset breast cancer who was diagnosed with bilateral high-grade invasive ductal carcinoma (luminal B, HER2-positive). She underwent bilateral skin- and nipple-sparing mastectomy with immediate reconstruction, followed by adjuvant chemotherapy and external-beam radiotherapy to the thoracic wall and right lymph node chains. Genetic testing subsequently confirmed a germline TP53 mutation consistent with LFS. Four years after irradiation, the patient developed a cutaneous angiosarcoma arising within the previously irradiated field.

This case illustrates a typical presentation of radiation-associated angiosarcoma in a genetically predisposed patient, with a relatively short latency period. While the temporal and spatial association with prior radiotherapy supports biological plausibility, the single-case design precludes definitive causal conclusions and underscores the ongoing clinical dilemma regarding radiotherapy use in LFS.

Across reported cases, secondary malignancies typically occurred within irradiated fields after relatively short latency periods, particularly in young patients (Table 1).

**Table 1:** Selected published case reports of secondary malignancies following radiotherapy in patients with Li-Fraumeni syndrome

First author / year	Primary cancer	RT indication	Radiation type	Secondary malignancy	Latency	Location relative to RT field
Kondapavulur et al., 2025 [16]	Low-grade astrocytoma	Adjuvant	Photon-based external-beam radiotherapy	Pleomorphic sarcoma (skull/soft tissue)	~4 years	Within irradiated field
Kondapavulur et al., 2025 [16]	Anaplastic glioma	Adjuvant	Proton therapy	Osteosarcoma of skull	~5 years	Within irradiated field
Barbosa et al., 2015 [17]	Invasive ductal breast carcinoma	Adjuvant (post–breast-conserving surgery)	Photon-based external-beam radiotherapy	Epithelioid angiosarcoma of the breast	~4–5 years	Within irradiated breast
García Novoa et al., 2019 [18]	Bilateral invasive ductal carcinoma (grade III, luminal B, HER2+)	Bilateral mastectomy + chemotherapy and external-beam radiotherapy	Photon-based external-beam radiotherapy	Radiation-induced cutaneous angiosarcoma of the right breast	4 years	Within the previously irradiated thoracic wall

Taken together, these case reports illustrate a recurring pattern that is biologically plausible but must be interpreted cautiously, as their descriptive nature and lack of comparator groups preclude definitive causal inference regarding radiotherapy-associated secondary cancer risk in LFS.

Retrospective Series

Retrospective evidence assessing the impact of radiotherapy in patients with LFS remains scarce and is derived from a limited number of pediatric and adult cohorts composed exclusively of germline TP53 mutation carriers. These studies are characterized by small sample sizes, heterogeneous indications, and variable follow-up durations.

The largest pediatric experience was reported by Woodward et al. [19], who retrospectively analyzed 47 children diagnosed with solid tumors before the age of 16, all carrying pathogenic germline TP53 variants. In this cohort, children treated with radiotherapy for their first malignancy developed second primary cancers earlier than those managed without radiotherapy. The median interval to a subsequent malignancy was 13.3 years in irradiated patients compared with 25.1 years in non-irradiated patients. Overall survival following the initial cancer diagnosis was also shorter among patients who received radiotherapy.

Adult breast cancer cohorts have reported heterogeneous but quantifiable rates of secondary malignancies following radiotherapy. In a single-institution retrospective chart review by Le et al. [20], including 94 patients with confirmed germline TP53 pathogenic variants, encompassing all histologic cancer types. Breast cancer represented the most frequent primary malignancy in this cohort. Among these patients, 20 received radiotherapy, of whom 18 were treated with curative intent. With a median follow-up exceeding 10 years, two of the 18 irradiated patients (11%) developed secondary malignancies attributable to prior radiotherapy, consisting of one sarcoma and one thyroid carcinoma, both arising within previously irradiated fields. Although the absolute number of radiation-induced malignancies was limited, the observed incidence exceeded that expected in unselected cancer populations, highlighting an increased susceptibility to treatment-related secondary cancers in TP53 mutation carriers.

Nandikolla et al. [21] described a small institutional series of four women with early-onset breast cancer and genetically confirmed LFS. All patients underwent surgery, and two received adjuvant external-beam radiotherapy before the underlying genetic predisposition was recognized. Over the course of follow-up, 2 patients developed additional primary malignancies (1 received RT, while the other one did not), including osteosarcoma in the patient who did not receive RT, and contralateral breast sarcoma in the patient who received RT. The interval to secondary cancer varied among patients, and secondary tumors did not consistently arise within irradiated fields, reflecting the intrinsic difficulty of separating treatment-related effects from the natural course of LFS.

Recent data were provided by Petry et al. [22] in a single-center retrospective cohort of breast cancer patients with pathogenic germline TP53 variants. Radiotherapy was administered to 30 of 48 patients (62%), and radiation-induced malignancies occurred in three patients (10%). Two were irradiated, arising within the irradiated field, while one patient has not been irradiated. No clear association was observed between radiation dose and secondary cancer risk, whereas radiotherapy technique appeared to influence outcomes, with secondary malignancies reported after two-dimensional and intensity-modulated radiotherapy but not after three-dimensional conformal techniques.

This study provides rare clinical data suggesting that radiotherapy technique may modulate secondary cancer risk in TP53 mutation carriers.

The largest adult cohort was reported by Sandoval et al. [23], who analyzed 227 women with early-stage breast cancer and germline TP53 pathogenic variants from international registries. Radiotherapy was administered to 79 patients (34.8%). Among irradiated patients, the five-year cumulative incidence of radiation-induced sarcoma was approximately 5%. Although lower than rates described in some smaller retrospective series, this incidence remains markedly higher than that observed in unselected breast cancer populations. Precise spatial correlation with radiation fields was not consistently detailed.

Across published pediatric and adult series, all reported patients were confirmed TP53 mutation carriers. Secondary malignancies occurred at variable frequencies, ranging from approximately 5% to over 70%, depending on cohort size, age at treatment, and duration of follow-up. When reported, secondary tumors frequently arose within irradiated fields, although distant malignancies were also observed, particularly in adult cohorts. Latency to secondary cancer ranged from several years to more than a decade, with shorter intervals consistently reported in irradiated pediatric patients. Taken together, these observations highlight the complexity of radiotherapy decision-making in LFS and support an individualized assessment of potential long-term risks and anticipated oncologic benefit (Table 2).

**Table 2:** Summary of selected retrospective cohorts evaluating secondary malignancies after radiotherapy in patients with Li-Fraumeni syndrome NR: not reported

Study	Population	TP53 status	RT exposure	Secondary malignancies	Latency to secondary cancer	Location of secondary tumors
Woodward et al. (2025) [19]	47 children (<16 y) with solid tumors	47/47 (100%)	22/47	20/47 (12RT vs 8 No RT)	Median 13.3 y (RT) vs 25.1 y (no RT)	9/12 in irradiation fields Vs 3/12 distant
Le et al. (2020) [20]	94 patients with different tumor sites	94/94 (100%)	20 (18 curative/2 palliative)	2/18 (11%)	4years (sarcoma) / 10years (Thyroid cancer)	2/18 in irradiation fields
Nandikolla et al. (2017) [21]	4 women with early-onset breast cancer	4/4 (100%)	2/4 (50%)	2/4 (50%) (1RT vs 1No RT)	NR	1/1 In-field sarcoma
Petry et al. (2024) [22]	48 women with breast cancer	48/48 (100%)	30/48 (62%)	3/30 (10%) (2RT 1 vs No RT)	3.9-16.5 years	2/2 in irradiation fields
Sandoval et al. (2024) [23]	227 women with early-stage breast cancer	227/227 (100%)	79/227 (34.8%)	6 (7.6%)	NR	6/6 sarcoma in the radiation field

The wide variability in reported rates of secondary malignancies across published case reports and retrospective cohorts should be interpreted with caution. Age at radiation exposure appears to be a key determinant, with pediatric patients consistently exhibiting shorter latency periods and higher cumulative risk compared with adults. Latency to secondary malignancy also varies substantially, ranging from a few years in children to more than a decade in adult cohorts, reflecting differences in tissue susceptibility, follow-up duration, and competing cancer risks inherent to LFS. Importantly, when spatial information is available, secondary tumors frequently arise within irradiated fields, supporting a biologically plausible association with radiation exposure, although distant malignancies are also observed, underscoring the intrinsic multi-cancer risk of germline TP53 mutation carriers. These factors collectively contribute to the heterogeneity of reported outcomes and preclude direct comparison across studies.

Taken together, available retrospective series are limited by small cohort sizes, heterogeneous clinical indications, and variable follow-up, preventing definitive risk quantification while still informing individualized clinical decision-making.

Is radiotherapy always contraindicated in LFS?

In clinical practice, radiotherapy in LFS should be restricted to carefully selected situations and decided within a multidisciplinary framework, given the increased susceptibility to radiation-induced carcinogenesis associated with germline TP53 pathogenic variants. Although avoidance of radiotherapy is generally recommended when curative alternatives exist, there are situations in which omission of local treatment may expose the patient to a high risk of disease progression or irreversible functional impairment.

Radiotherapy may be justified in the presence of microscopically or macroscopically positive surgical margins (R1 or R2) that cannot be addressed by further resection, particularly when residual disease threatens durable local control. Similarly, tumors deemed inoperable because of anatomical constraints or unacceptable surgical morbidity may necessitate radiotherapy as the only viable local treatment option. In functional emergencies, such as spinal cord compression, airway compromise, or vascular obstruction, timely radiotherapy can be essential to prevent permanent neurological or organ damage. Radiotherapy also retains a role in palliative settings, where symptom relief-such as pain, bleeding, or mass effect-is the primary therapeutic goal. Finally, radiotherapy may be considered when there is an absence of effective systemic therapeutic alternatives, especially in tumors with limited chemosensitivity or in patients who have exhausted available systemic options [7,22].

In all these contexts, the decision to deliver radiotherapy to patients with LFS must be made through a multidisciplinary discussion, integrating surgical, medical, radiation oncology, and genetic expertise, as well as patient preferences. Whenever feasible, alternative local treatments should be prioritized to ensure that the anticipated oncologic or functional benefit clearly outweighs the potential long-term risk of secondary malignancy [24].

When radiotherapy is considered: how can risk be mitigated?

When radiotherapy is deemed unavoidable in patients with LFS, meticulous treatment planning is essential to reduce the risk of radiation-associated secondary malignancies. Given the intrinsic radiosusceptibility associated with germline TP53 pathogenic variants, every effort should be made to limit unnecessary exposure of normal tissues while preserving therapeutic efficacy [22].

Reducing Irradiated Volumes

One pragmatic strategy that has been proposed in this context is the reduction of irradiated volumes. When radiotherapy is deemed unavoidable, target delineation may be restricted to areas of gross disease or regions at highest risk of microscopic involvement, while avoiding prophylactic or elective nodal irradiation whenever this is considered oncologically acceptable [22]. Such a volume-sparing approach is based on the rationale of limiting the exposure of normal tissues to ionizing radiation and, consequently, the cumulative mutational burden in radiosensitive cell populations, particularly in the setting of impaired DNA damage surveillance [25].

Impact of Modern Photon Techniques

The use of highly conformal radiotherapy techniques, such as volumetric modulated arc therapy (VMAT), is increasingly favored over older two-dimensional (2D), three-dimensional conformal radiotherapy (3D-CRT), and even static intensity-modulated radiotherapy (IMRT) approaches in patients with LFS when irradiation is unavoidable. Compared with historical 2D techniques, which rely on large, geometrically simple fields and inevitably expose substantial volumes of normal tissue, VMAT allows a marked reduction in unnecessary irradiation outside the target volume. Relative to 3D-CRT, which improves target coverage but still delivers significant intermediate and high doses to adjacent normal tissues, VMAT offers superior dose conformity and sharper dose gradients around critical organs at risk [22].

When compared with static IMRT, VMAT achieves similar or improved target coverage while typically requiring fewer monitor units and shorter delivery times [26]. This reduction in monitor units may translate into lower scatter radiation and fewer unintended micro-dose exposures to surrounding normal tissues, an aspect of particular relevance in patients with germline TP53 mutations who exhibit impaired DNA damage surveillance. Nevertheless, VMAT, like other highly modulated techniques, may increase the volume of tissue receiving low-dose radiation, the so-called “low-dose bath.” Given the heightened susceptibility to radiation-induced malignancies and the young age at treatment of many Li-Fraumeni patients, careful attention to low-dose exposure remains essential, as the long-term implications of such exposure in TP53 mutation carriers are not yet fully defined [24].

Early modeling studies suggested a potentially increased risk of secondary malignancies with IMRT compared with 3D-CRT, although these estimates were frequently based on high photon energies no longer routinely used. Clinical data remain conflicting, with some studies reporting modest differences between techniques and others showing no significant excess risk, likely reflecting limited follow-up and complex dose-volume relationships [27]. In patients with heritable TP53-associated cancer syndromes, evidence is insufficient to support firm technique-specific recommendations. Therefore, photon radiotherapy modality selection should be individualized, balancing tumor coverage against minimization of normal tissue exposure in the context of presumed increased radiosusceptibility.

In summary, while modern highly conformal photon techniques such as VMAT may reduce unnecessary high-dose exposure compared with older approaches, their potential impact on low-dose irradiation remains an important consideration in patients with LFS. Consequently, photon technique selection should be individualized, with careful balancing of target coverage and normal tissue sparing in the context of presumed increased radiosusceptibility.

Proton Therapy

Proton therapy is a form of external beam radiotherapy that uses charged particles rather than photons and is distinguished by its characteristic depth-dose distribution. As protons traverse tissue, they gradually lose kinetic energy and deposit most of their dose at a finite depth, known as the Bragg peak. By modulating this peak to generate a spread-out Bragg peak, proton therapy allows coverage of the full tumour thickness while delivering minimal dose beyond the target volume, thereby reducing irradiation of distal normal tissues compared with photon-based techniques [24].

From a dosimetric perspective, proton therapy generally results in lower integral dose and smaller irradiated normal tissue volumes than conventional photon radiotherapy, including intensity-modulated techniques. Modelling studies that incorporate parameters such as cell killing, mutation induction, repopulation, and heterogeneous organ dose distributions have consistently estimated a lower probability of radiation-induced second malignant neoplasms with proton therapy than with intensity-modulated photon radiotherapy, often by a factor of several [28,29]. These differences are primarily attributed to physical volume-sparing effects rather than to fundamental differences in biological effectiveness.

Proton therapy can achieve homogeneous target dose distributions with a limited number of beam angles, although the magnitude of normal tissue sparing may decrease as the number of fields increases. Additional reductions in irradiated volume may be possible with partial-organ irradiation strategies when clinically appropriate. From a technical standpoint, active delivery methods such as pencil beam scanning are generally preferred over passive scattering techniques, as the latter are associated with increased production of secondary neutrons, which may partially offset the dosimetric advantages of protons [26].

Although protons are classified as low linear energy transfer radiation, linear energy transfer increases toward the distal edge of the proton beam. Correspondingly, relative biological effectiveness values have been reported to increase from approximately 1.1 in the entrance region to higher values in the distal fall-off [29,30]. Higher linear energy transfer is associated with denser ionisation patterns and more complex DNA damage. In patients with heritable TP53-related cancer syndromes, alterations in DNA damage responses have been described, including delayed rejoining of DNA double-strand breaks reflecting an altered damage response threshold required to trigger cell-cycle arrest [31].

In a large retrospective analysis of the National Cancer Database of over 450,000 patients [28], proton beam radiotherapy was associated with a significantly lower risk of second primary malignancies compared with intensity-modulated photon radiotherapy. This reduction persisted after multivariable adjustment and propensity score matching across multiple tumour sites. These findings suggest a potential advantage of proton therapy in limiting radiation-induced carcinogenic risk through reduced normal tissue irradiation.

Finally, proton therapy is more sensitive than photon radiotherapy to uncertainties in treatment planning and delivery, including organ motion, respiratory effects, and the presence of implanted materials such as metallic prostheses within or near the treatment field. These factors require careful evaluation by experienced medical physicists. Overall, when radiotherapy is unavoidable, proton therapy appears to offer a favourable balance between tumour coverage and reduction of normal tissue exposure in patients with heritable TP53-related cancer syndromes, although long-term risks remain incompletely defined [24].

Collectively, these strategies underscore the importance of individualized radiotherapy planning in LFS. When radiotherapy is unavoidable, treatment should be delivered using the most advanced and conservative techniques available, within a multidisciplinary framework, to minimize long-term risk while addressing immediate oncologic needs. Despite its favorable dosimetric properties, long-term safety data for proton therapy in TP53 mutation carriers remain limited, and its use should therefore be guided by availability, institutional expertise, and individualized risk assessment.

Post-treatment surveillance and long-term follow-up

Follow-up after radiotherapy in LFS should be more intensive than in non-hereditary settings, given the cumulative genetic risk and documented occurrence of second malignancies. Surveillance strategies for germline TP53 mutation carriers typically include annual whole-body MRI, organ-specific imaging (e.g., breast MRI beginning at a young age for women), and regular clinical assessment to identify new or recurring tumors early. These protocols also support field-specific monitoring of previously irradiated areas to facilitate prompt detection of RIM. Minimizing additional genotoxic imaging, where possible, and coordinating follow-up within multidisciplinary hereditary cancer programs can further optimize long-term care [32].

In summary, long-term follow-up represents a cornerstone of care in LFS, particularly after radiotherapy, to support early detection of subsequent malignancies while limiting additional genotoxic exposure.

Toward a personalized decision-making framework

In LFS, radiotherapy cannot be guided by uniform recommendations. Germline TP53 pathogenic variants may increase susceptibility to radiation-associated malignancies, while available clinical data remain limited and heterogeneous. Treatment decisions should therefore rely on individualized risk-benefit assessment rather than categorical avoidance or routine use of radiotherapy [24].

Radiotherapy should be avoided when effective non-radiation alternatives exist, particularly in curative settings. However, it may be justified in selected situations, such as unresectable disease, positive surgical margins not amenable to further resection, functional emergencies, or palliative indications where local control or symptom relief is essential. When radiotherapy is considered necessary, efforts should focus on minimizing normal tissue exposure through careful target volume selection and the use of highly conformal techniques [7,22].

All decisions should be made within a multidisciplinary framework and incorporate patient preferences. When radiotherapy is delivered, intensified long-term surveillance is required to facilitate early detection of subsequent malignancies [32].

Overall, radiotherapy in LFS should be considered on a case-by-case basis, with careful balancing of anticipated oncologic benefit against uncertain long-term risks in the absence of prospective data.

## Conclusions

LFS poses specific challenges for the use of radiotherapy, as germline TP53 pathogenic variants may modify cellular responses to DNA damage and potentially increase susceptibility to radiation-associated malignancies. Current evidence is predominantly retrospective and heterogeneous, limiting definitive conclusions regarding causality and the magnitude of risk. Until prospective data become available, radiotherapy should remain a carefully selected option, considered when the potential oncologic or functional benefit outweighs the uncertain long-term risks, and decided through multidisciplinary discussion. When used, efforts to limit irradiated volumes and employ highly conformal techniques appear reasonable. Proton therapy may offer additional dosimetric advantages, although its long-term safety in TP53 mutation carriers remains incompletely defined. Prospective studies and international collaborative registries are needed to better clarify the role of radiotherapy in this rare hereditary cancer syndrome.
